# Efficacy and Safety of Fluorescence-Guided Surgery Compared to Conventional Surgery in the Management of Colorectal Cancer: A Systematic Review and Meta-Analysis

**DOI:** 10.3390/cancers16193377

**Published:** 2024-10-02

**Authors:** Michael G. Fadel, Elham Zonoobi, María Rita Rodríguez-Luna, Kohei Mishima, Frédéric Ris, Michele Diana, Alexander L. Vahrmeijer, Silvana Perretta, Hutan Ashrafian, Matyas Fehervari

**Affiliations:** 1 Department of Surgery and Cancer, Imperial College London, London SW7 2AZ, UK; 2 Department of General Surgery, Chelsea and Westminster Hospital, London SW10 9NH, UK; 3 Edinburgh Molecular Imaging Limited, Nine Edinburgh Bioquarter, Edinburgh EH16 4UX, UK; 4 Department of Surgery, Leiden University Medical Center, 2333 ZA Leiden, The Netherlands; 5 Department of General Surgery, Hospital de Barcelona, 08001 Barcelona, Spain; 6 Research Institute Against Digestive Cancer (IRCAD), 67000 Strasbourg, France; 7 Department of Surgery, University Hospital of Geneva, 1205 Geneva, Switzerland; 8 Faculty of Medicine, University of Geneva, 1211 Geneva, Switzerland; 9 ICube Laboratory, Photonics Instrumentation for Health, 67034 Strasbourg, France; 10 IHU-Strasbourg, Institute of Image-Guided Surgery, 67000 Strasbourg, France; 11 Department of Digestive and Endocrine Surgery, University of Strasbourg, 67081 Strasbourg, France; 12 Department of Gastrointestinal Surgery, Maidstone and Tunbridge Wells NHS Trust, Tunbridge Wells TN2 4QJ, UK

**Keywords:** colorectal cancer, fluorescence-guided surgery, indocyanine green, efficacy, safety, anastomotic leakage

## Abstract

**Simple Summary:**

Colorectal cancer is the second highest cause of cancer mortality globally. Surgery is often performed to remove the cancer and improve survival. Special dyes, called fluorescent agents, can help surgeons identify the tumour tissue and guide them during the operation to precisely remove the cancer, thereby improving patient outcomes. The exact benefit of fluorescence-guided surgery in the management of colorectal cancer is not clearly established, necessitating this systematic review and meta-analysis of the literature to assess the safety and efficacy of this surgery. A total of 35 studies of 3217 patients were included in this analysis. This showed that fluorescence-guided surgery is a safe and effective approach, potentially reducing intraoperative blood loss and postoperative complication rates, when compared to surgery without the use of fluorescence agents. Further prospective clinical trials are required to establish the long-term oncological benefit of fluorescence-guided surgery.

**Abstract:**

Background: The use of fluorescence agents and imaging systems is a promising adjunct in the surgical management of colorectal cancer. This systematic review and meta-analysis aimed to assess the safety and efficacy of fluorescence-guided surgery in the management of colorectal cancer, with a comparison to conventional (non-fluorescence-guided) surgery. Methods: A literature search of MEDLINE, Embase, Emcare, and CINAHL databases was performed for studies that reported data on the outcomes of fluorescence-guided surgery, with or without a comparison group undergoing conventional surgery, for colorectal cancer between January 2000 and January 2024. A meta-analysis was performed using random-effect models, and between-study heterogeneity was assessed. Results: 35 studies of 3217 patients with colorectal cancer were included: 26 studies (964 patients) reported on fluorescence-guided surgery and 9 studies (2253 patients) reported on fluorescence versus conventional surgery. The weighted mean of the cancer detection rate of fluorescence-guided surgery was 71% (95% CI 0.55–0.85), with no significant difference in lymph node yield ratio (WMD −0.04; 95% CI −0.10–0.02; *p* = 0.201) between fluorescence and conventional surgery groups. There was a significantly lower blood loss (WMD −4.38; 95% CI −7.05–−1.70; *p* = 0.001) and complication rate (WMD −0.04; 95% CI −0.07–0.00; *p* = 0.027) in the fluorescence-guided surgery group, with a potentially lower anastomotic leak rate (WMD −0.05; 95% CI −0.10–0.01; *p* = 0.092). Conclusions: Fluorescence-guided surgery is a safe and effective approach in the management of colorectal cancer, potentially reducing blood loss and complications. Further randomised controlled trials are required comparing fluorescence-guided surgery with conventional surgery to determine its prognostic benefit and where it should precisely fit within the management pathway of colorectal cancer.

## 1. Introduction

Colorectal cancer (CRC) imposes a significant global health burden, with escalating incidence rates worldwide [1,2]. Several factors, including genetic, immune, and environmental factors, are known to influence the development of CRC. Changes in lifestyle, especially dietary patterns, and gut microbiota modulation have been shown to greatly affect the progression of CRC [3,4,5]. Nevertheless, surgical intervention remains pivotal in patients with CRC, with complete tumour resection leading to improved overall survival and lower recurrence rates [6,7,8]. Despite recent improvements in mortality rates attributed to screening initiatives, neoadjuvant therapies, and surgical techniques, surgeons still face challenges in distinguishing between benign and malignant tissue intraoperatively. This reliance on subjective tactile and visual assessments can lead to inaccuracies in assessing tumour margins, resulting in positive resection margins [9,10]. The occurrence of positive margins can contribute to local recurrence and metastases within a short timeframe, suggesting potential oversight of tumours in previous evaluations [11].

Near-infrared fluorescence (NIRF) imaging is rapidly growing in the clinical setting to help surgeons make intraoperative assessments and decisions. It has advantages, including real-time visualisation, high spatial resolution, no ionising radiation, and portability. NIRF imaging enhances the visualisation of normal and tumour tissue by exploiting the deep tissue penetration capability of NIRF photons (up to 5 to 10 mm) [12]. Fluorophores, accumulating in tumours either through enhanced permeability and retention or by targeting specific markers, emit NIRF light when activated by a laser, light-emitting diode, or Xenon. Specialised imaging systems capture this emitted light, allowing NIRF-guided surgery to differentiate between benign and malignant tissues. Lesions beyond can standard resection fields can be detected, along with lymphatic networks and pathways of bowel perfusion to prevent anastomotic leaks [13].

NIRF agents are usually administered intravenously and can be categorised into two main groups: tumour-specific-targeted and non-targeted fluorescent contrast agents. Indocyanine green (ICG) is the most frequently used non-targeted fluorescent agent in NIRF imaging. It is a fluorescent water-soluble dye that binds with plasma proteins, especially lipoproteins, and demonstrates fluorescent properties in the NIRF spectrum (750–950 nm) [12]. On the other hand, tumour-targeted fluorescence-guided surgery uses NIRF probes that target moieties, such as antibodies or ligands, or bind to proteins or receptors overexpressed on tumour cells, emitting NIRF light. A variety of targeted fluorescent agents are currently under evaluation in phase I–III clinical trials, indicating a dynamic evolution in imaging techniques [14,15].

This systematic review and meta-analysis provides a comprehensive assessment of the role of fluorescence-guided surgery in CRC surgery, with a comparison to conventional (non-fluorescence-guided) surgery. Efficacy outcomes will be presented, including cancer detection rate and lymph node yield ratio. We will also present surgical and safety outcomes, including operative time, estimated blood loss, length of stay, and perioperative complications, including anastomotic leak rate.

## 2. Methods

### 2.1. Search Strategy

A literature search of MEDLINE, Embase, Emcare, and CINAHL databases was performed (Supplementary Table S1). For each database, specific research equations were created using Medical Subject Headings (MeSH) terms, such as fluorescence, near-infrared imaging, medical optical imaging, endoscopy, fluorescence-guided surgery, fluorescence imaging, indocyanine green, colorectal cancer, colorectal tumour, and colorectal neoplasm. We retrieved articles published in the English language between 1 January 2000 and 1 January 2024 that assessed the efficacy and safety of fluorescence-guided surgery in the management of CRC. We also manually reviewed the references from the identified studies to select any further relevant studies.

This study was conducted using the Preferred Reporting Items for Systematic Reviews and Meta-Analysis (PRISMA) guidelines [16] and the Cochrane Handbook for Systematic Reviews of interventions [17], and it was registered in the PROSPERO database in May 2023 (CRD42023424400).

### 2.2. Study Selection and Data Extraction

All studies reporting on clinical outcomes of fluorescence-guided surgery or comparing outcomes of fluorescence-guided surgery versus conventional surgery in the management of CRC (including metastatic disease) were eligible. Randomised controlled trials (RCTs), prospective, and retrospective studies were included in this review. The following exclusion criteria were applied: (i) fluorescence-guided surgery in the management of benign lesions, (ii) conference abstracts, case reports, editorial letters, book chapters, or duplicated data, (iii) articles published in a non-English language, and (iv) animal studies.

Two authors (MGF and EZ) performed the search independently using the inclusion and exclusion criteria, with any disagreement resolved by a third independent reviewer (MRRL). The full-text article was subsequently read to decide whether the article should be included in the analysis. We extracted the following from each article: first author, publication year, country, design of study, sample size, age, sex, duration of follow-up, inclusion and exclusion criteria, tumour staging (T1–T4), type of fluorescent agent, dose and mode of administration, molecular target (if applicable), imaging device, and time between injection and imaging. The primary outcomes extracted included number of lymph nodes harvested, number of positive lymph nodes harvested, lymph node yield ratio, and the cancer detection rate. The cancer detection rate was defined as the number of malignant lesions divided by the total number of lesions excised. Secondary outcomes extracted include tumour size (cm), estimated blood loss (mL), operative time (minutes), and complication rates, including anastomotic leakage.

### 2.3. Quality Assessment of Studies

The quality of all observational studies was independently assessed by two authors (MGF and EZ) using the Newcastle–Ottawa Scale (NOS) [18], with any differences resolved by discussion and consensus. The NOS was calculated according to the method of patient selection, comparability of the study groups, and assessment of outcomes reported. The highest possible score was nine stars, and studies that had a score of seven or higher were deemed to be of high quality. The strength of clinical data and subsequent recommendations of all studies were also graded according to the Oxford Centre for Evidence-Based Medicine [19] by one author (MGF): level 1, evidence from a systematic review/meta-analysis and RCT; level 2, controlled trial with no randomisation or a prospective study; level 3, retrospective cohort or case-control study.

### 2.4. Statistical Analysis

The weighted mean difference (WMD) and the logarithm of DerSimonian–Laird (DL) with 95% confidence intervals (CI) was calculated. A *p*-value of ≤0.05 was deemed to be statistically significant. Between-study heterogeneity was assessed using the *I*^2^ value to measure the degree of variation not attributable to chance alone. This was graded as low (*I*^2^ < 25%), moderate (*I*^2^ = 25–75%), or high (*I*^2^ > 75%). The meta-analysis of data was conducted using a random-effects model, and all statistical analyses, including the forest plots, were performed using Reviewer Manager 5.4.1 software.

## 3. Results

**Figure 1:**
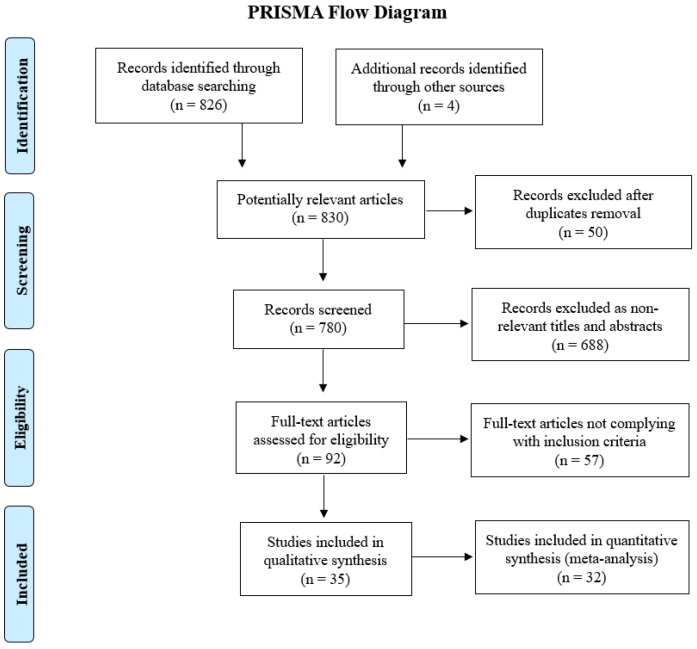
The flowchart shows the literature search and study selection process according to the PRISMA guidelines.

The initial database search and additional records identified 830 publications. A total of 738 articles were excluded after title and abstract review and removal of duplicates. Thus, 92 articles were fully reviewed, and 35 and 32 studies were included in the qualitative and quantitative analysis, respectively. A total of 3217 CRC patients were included in this systematic review. Here, 26 studies (964 patients) reported data on fluorescence-guided surgery only, and 9 studies (2253 patients) presented data on fluorescence versus conventional (non-fluorescence-guided) surgery. The PRISMA diagram of the literature search is displayed in Figure 1.

### 3.1. Baseline Study and Patient Characteristics

The baseline study and patient characteristics and the quality assessment of the included studies are summarised in Table 1. Of the 35 studies [20,21,22,23,24,25,26,27,28,29,30,31,32,33,34,35,36,37,38,39,40,41,42,43,44,45,46,47,48,49,50,51,52,53,54], 9 were retrospective [22,28,40,42,43,44,48,49,53], 21 studies [20,21,23,24,25,26,27,29,31,32,33,34,35,36,38,39,46,47,50,51,52] were prospective, and 5 were RCTs [30,37,41,44,54]. The NOS score was applied, as the majority of the studies were observational studies, and 22 out of the 30 observational studies were considered to be of low risk of bias (Supplementary Table S2).

Eleven studies were from Japan and nine studies were from The Netherlands, with the remaining studies from China, Germany, Italy, Korea, Switzerland, the UK, and the USA. The median or mean ages ranged from 55.7 years to 75.0 years from the included studies. The tumour stage was reported in 15 studies, with 484/1224 (39.5%) with T1–T2 disease and 740/1224 (60.5%) with T3–T4 disease. The inclusion and exclusion criteria of the studies for fluorescence-guided surgery are presented in Supplementary Table S3. ICG was the most commonly used agent (25 studies), followed by the antibody-dye conjugate SGM-101 (2 studies). The most common route of injection was intravenous, and the time from injection to imaging ranged from immediate to 5–7 days. The median or total follow-up ranged from immediately postoperatively to 4 years across the studies.

### 3.2. Fluorescence-Guided Surgery

#### 3.2.1. Surgical and Safety Outcomes

**Figure 2:**
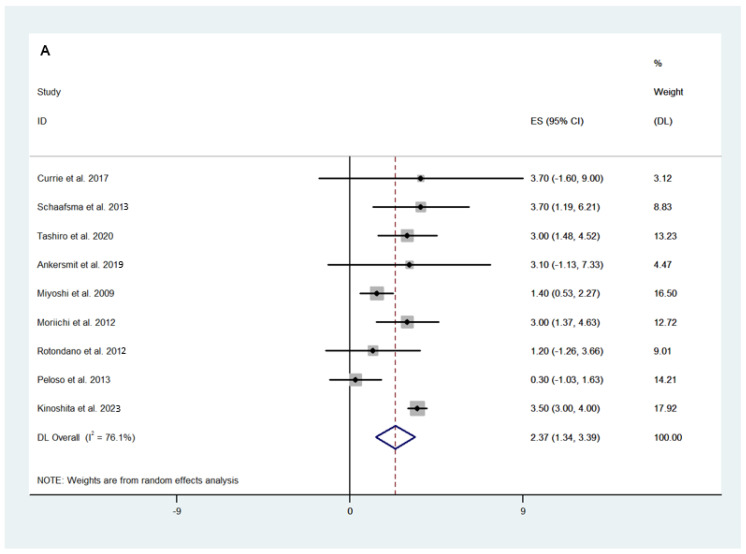

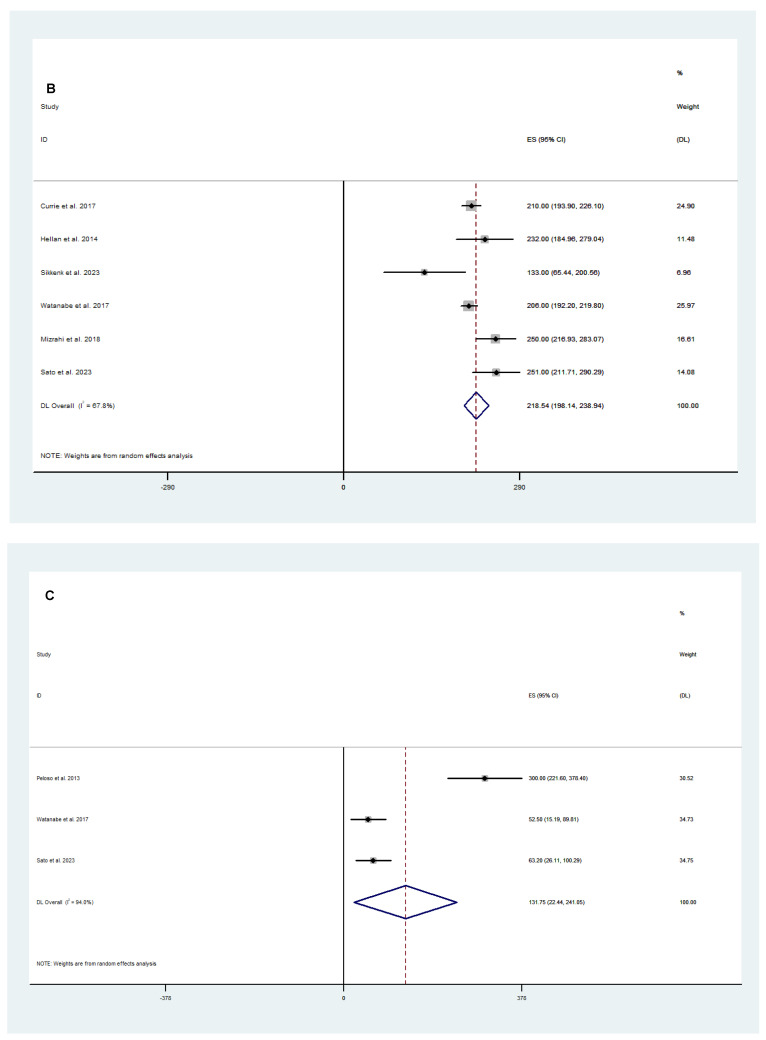

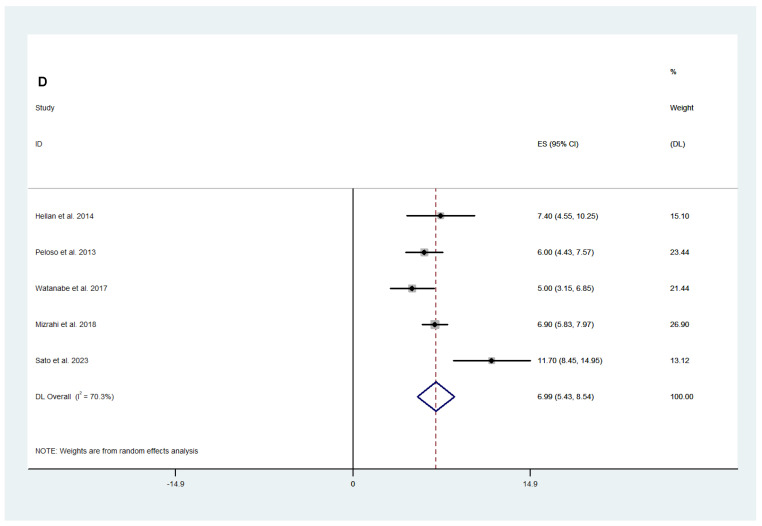
Weighted mean of (**A**) tumour size, (**B**) operative time, (**C**) estimated blood loss, and (**D**) length of stay following fluorescence-guided surgery [21,23,25,26,32,34,35,36,37,38,41,46,48,51].

In 9 studies, with a total of 369 patients, the tumour size was reported prior to fluorescence-guided surgery. The pooled analysis of these studies revealed that the weighted mean of the tumour size was 2.37 cm (95% CI 1.34–3.39; *I*^2^ = 76.1; Figure 2A). The weighted mean operative time of fluorescence-guided surgery was 218.5 min (95% CI 198.14–238.94; *I*^2^ = 67.8%) in 179 patients across 6 studies (Figure 2B). Only 3 studies (70 patients) reported estimated intraoperative blood loss, for which the weighted mean was 131.75 mL (95% 22.44–241.05; *I*^2^ = 94.0%) (Figure 2C). The mean of the postoperative length of stay was 6.99 days (95% 5.43–8.54; *I*^2^ = 70.3%) amongst 5 studies in 142 patients (Figure 2D).

**Figure 3:**
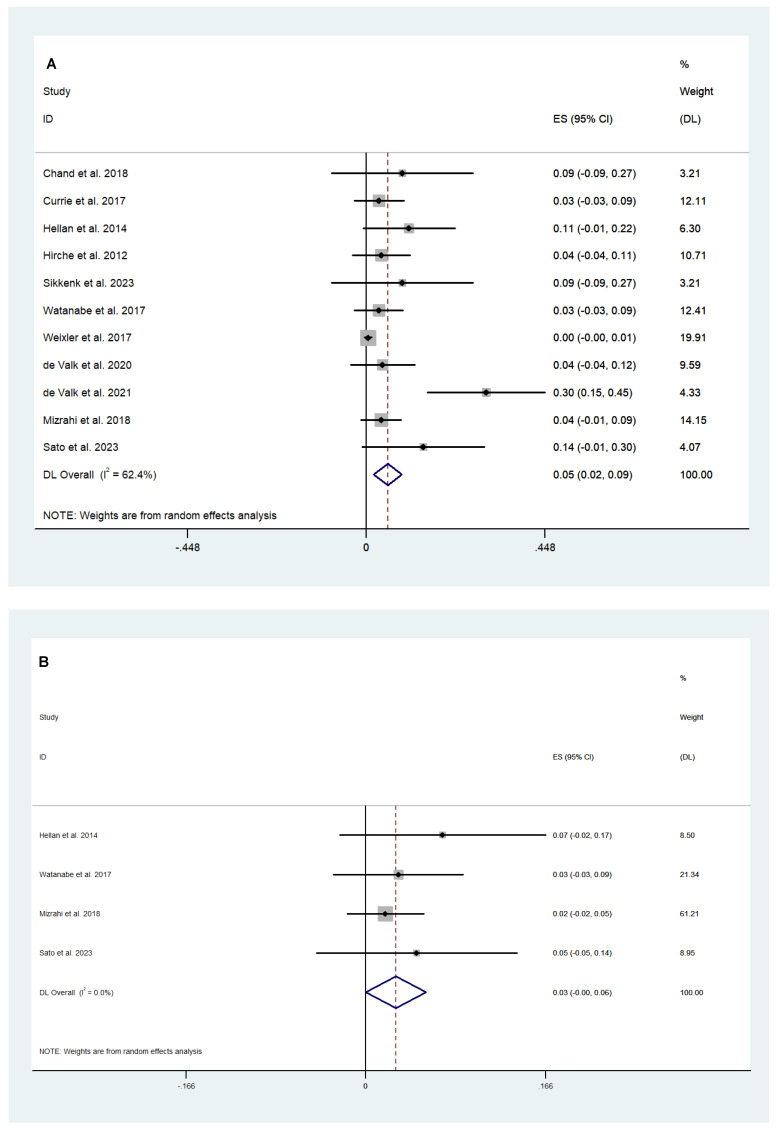
Weighted mean of (**A**) complications and (**B**) anastomotic leak rate following fluorescence-guided surgery [20,21,23,24,26,28,42,44,47,48,51].

A total of 567 patients, from 11 studies, reported postoperative complication rates following fluorescence-guided surgery. The complication rate was 5% (95% CI 2.00–9.00; *I*^2^ = 62.4%; Figure 3A), which included postoperative ileus, urinary retention, surgical site infection, pleural effusion, pelvic fluid collection, deep venous thrombosis, and minor intra-abdominal bleeding. There was a 3% anastomotic leak rate (95% CI 0.00–6.00; *I*^2^ = 0.0%) reported in 4 studies of 139 patients (Figure 3B). There were no adverse events or anaphylactic reactions related to fluorescence agents across all studies.

#### 3.2.2. Efficacy Outcomes

**Figure 4:**
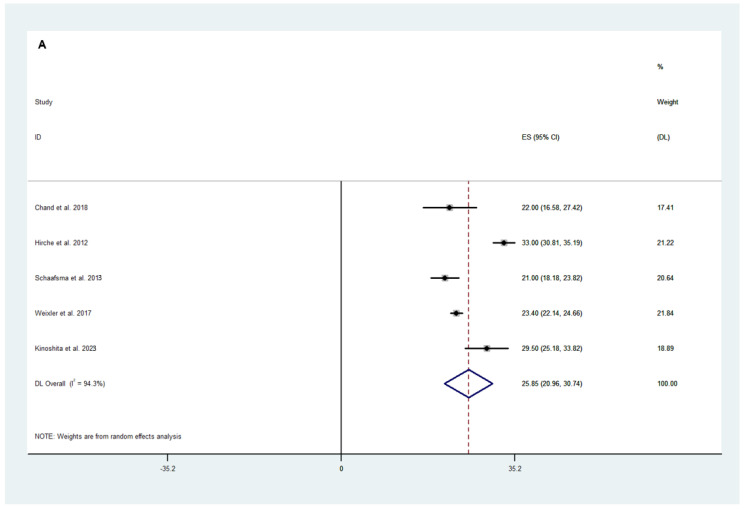

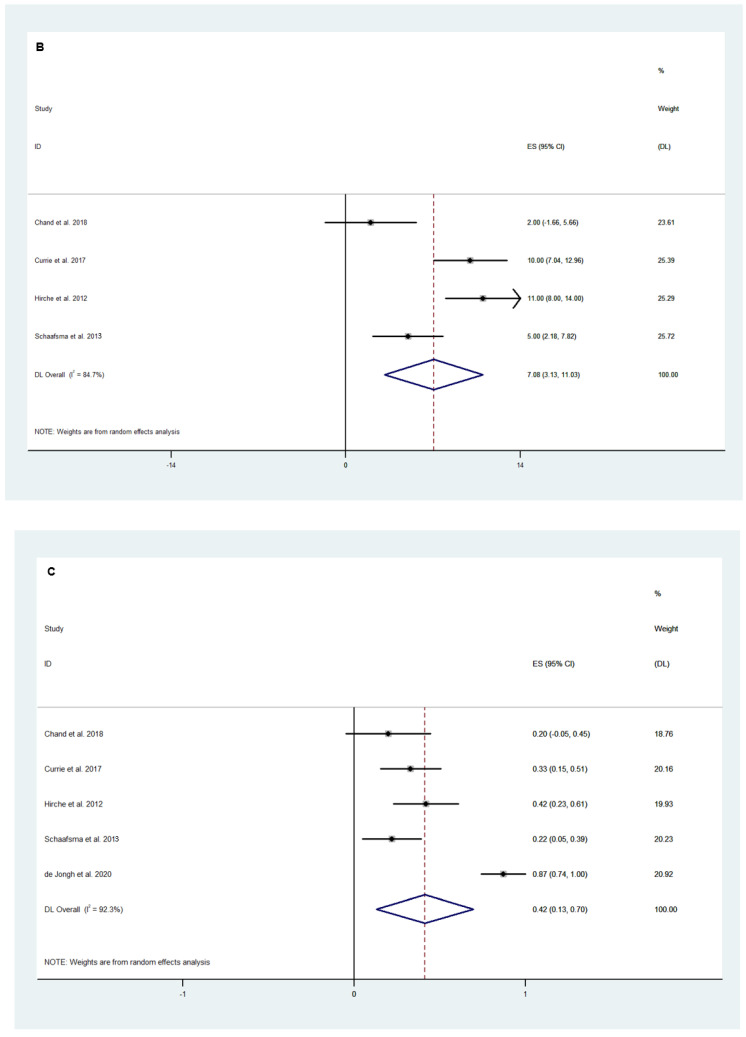

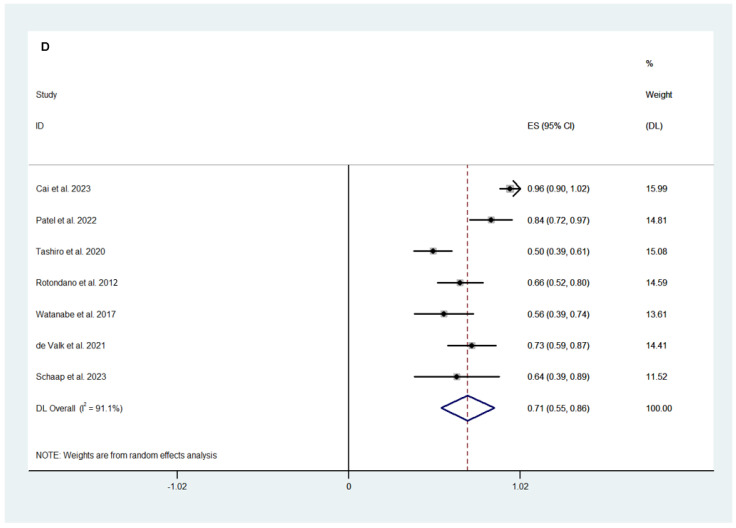
Weighted mean of (**A**) number of lymph nodes harvested, (**B**) number of positive lymph nodes harvested, (**C**) lymph node yield ratio, and (**D**) cancer detection rate following fluorescence-guided surgery [20,21,24,25,29,31,32,37,41,42,43,46,47,52].

In 5 studies, covering 361 patients, the number of lymph nodes harvested was reported. The weighted mean of lymph nodes harvested was 25.85 (95% CI 20.96–30.74; *I*^2^ = 94.3%; Figure 4A), and positive lymph nodes harvested was 7.08 (95% CI 3.13–11.03; *I*^2^ = 84.7%; Figure 4B), with a lymph node yield ratio of 42% (95% CI 13.00–70.00; *I*^2^ = 92.3%; Figure 4C). The pooled analysis of the cancer detection rate was 71% (95% CI 55.00–85.00; *I*^2^ = 91.1%) across 7 studies of 281 patients (Figure 4D). Four of these studies used ICG, with the other studies using SGM-101 to detect CRC, ranging from 30 min to seven days prior to surgery. Long-term oncological outcomes were only recorded by Weixler et al. [42]. In 220 CRC patients undergoing fluorescence-guided surgery, the 3-year and 5-year overall survival was 86% and 75%, respectively, and the 3-year and 5-year disease-free survival was 88% and 82%, respectively.

### 3.3. Fluorescence-Guided versus Conventional Surgery

#### Safety and Efficacy Outcomes

**Figure 5:**
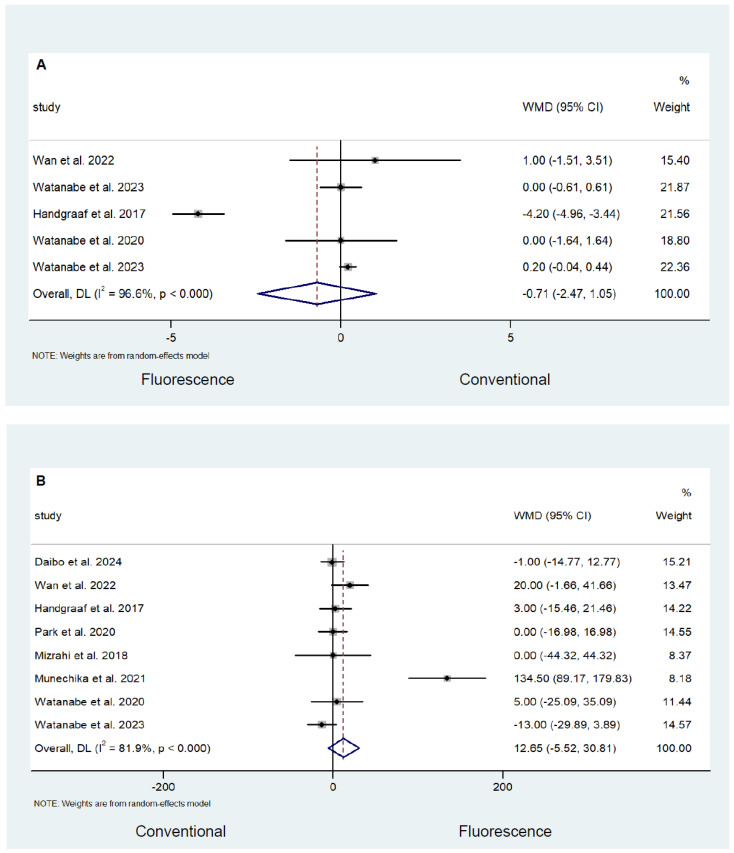

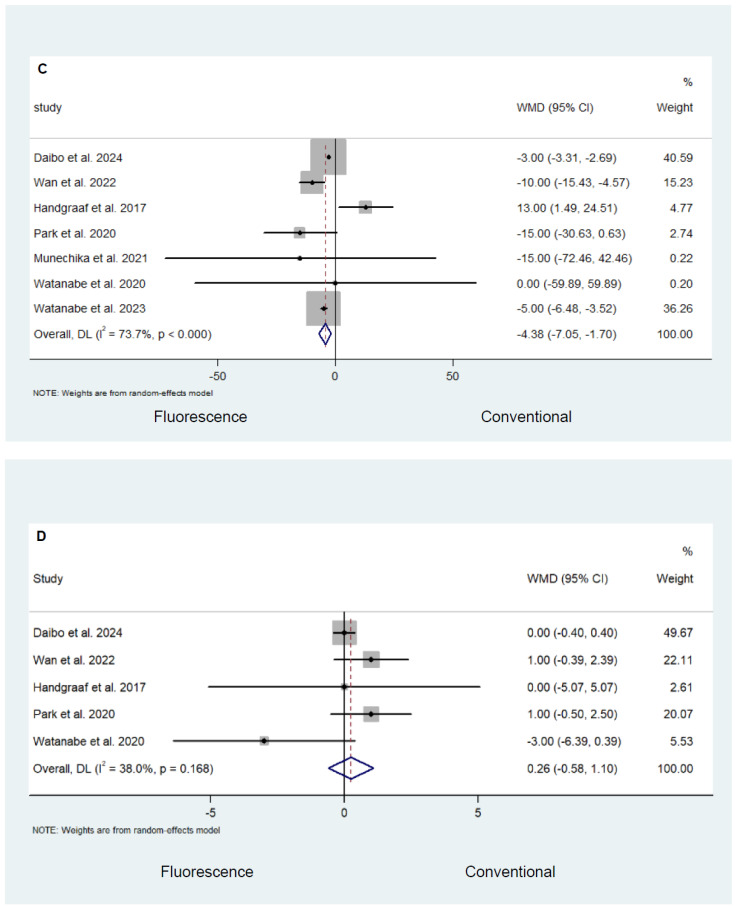
Comparison of (**A**) tumour size, (**B**) operative time, (**C**) estimated blood loss, and (**D**) length of stay following fluorescence-guided surgery and conventional surgery [22,27,28,40,45,49,50,53,54].

There was no significant difference in tumour size between the fluorescence and conventional surgery groups (WMD −0.71; 95% CI −2.47–1.05; *p* = 0.431; *I*^2^ = 96.6%; Figure 5A) and operative time (WMD 12.65; 95% CI −5.52–30.81; *p* = 0.172; *I*^2^ = 81.9%; Figure 5B). The fluorescence-guided surgery had a lower estimated blood loss (WMD −4.38; 95% CI −7.05–−1.70; *p* = 0.001; *I*^2^ = 73.7%; Figure 5C); however, there was no difference in length of stay (WMD 0.26; 95% CI −0.58–1.10; *p* = 0.550; *I*^2^ = 38.0%; Figure 5D) between the two groups.

**Figure 6:**
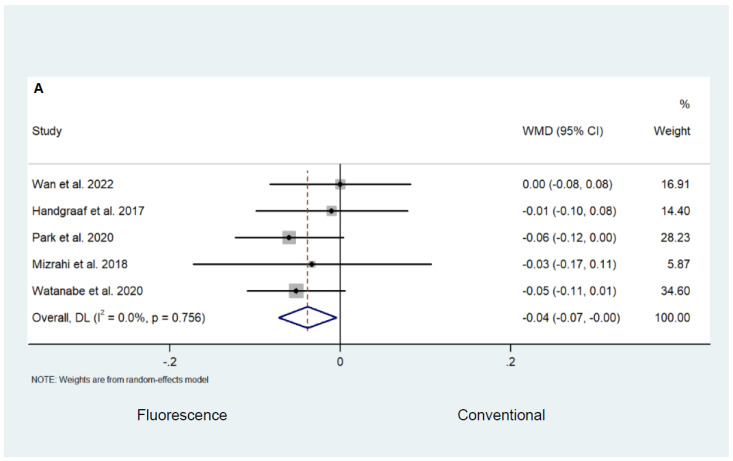

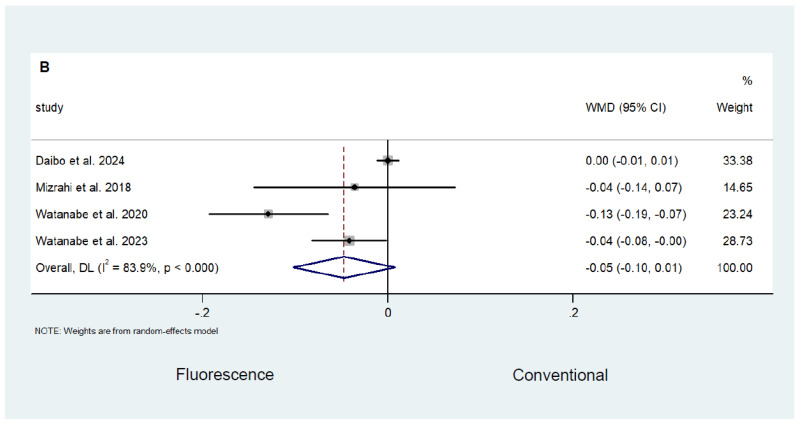
Comparison of (**A**) complications (Clavien–Dindo classification grade 3 or higher) and (**B**) anastomotic leak rate following fluorescence-guided surgery and conventional surgery [22,27,40,45,49,53,54].

**Figure 7:**
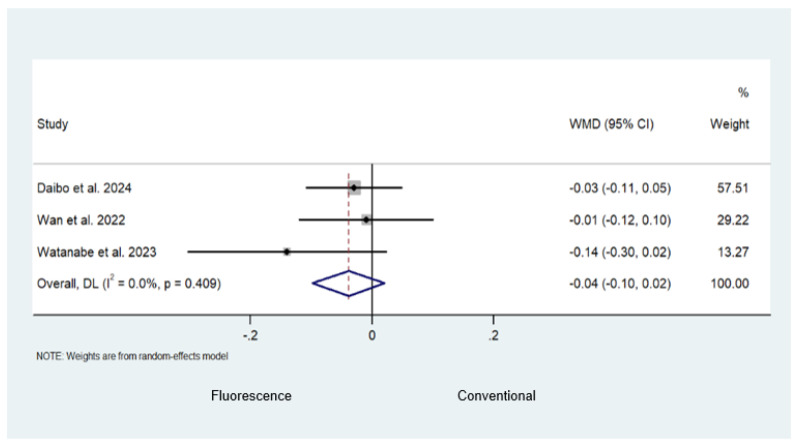
Comparison of the lymph node yield ratio following fluorescence-guided surgery and conventional surgery [22,27,54].

Random-effect analysis revealed a lower complication rate (Clavien–Dindo Classification grade 3 or higher) in the fluorescence-guided surgery group compared to the conventional surgery group (WMD −0.04; 95% CI −0.07–0.00; *p* = 0.027; *I*^2^ = 0.0%; Figure 6A). Although not statistically significant, there was some evidence found to suggest that fluorescence-guided surgery had a lower anastomotic leak rate when compared to conventional surgery (WMD −0.05; 95% CI −0.10–0.01; *p* = 0.092; *I*^2^ = 83.9%; Figure 6B). Pooled analysis revealed that fluorescence-guided surgery was not superior to conventional surgery in terms of the lymph node yield ratio (WMD −0.04; 95% CI −0.10–0.02; *p* = 0.201; *I*^2^ = 0.0%; Figure 7). Long-term oncological outcomes were only reported by two studies. Daibo et al. [22] reported a similar three-year overall survival of 94.5% in the fluorescence-guided surgery group versus 94.7% in the conventional surgery group. The three-year recurrence-free survival was reported as 88.8% and 89.4% in the fluorescence and conventional surgery groups, respectively. Watanabe et al. [28] demonstrated a 93.1% and 85.9% in three-year overall survival and a three-year recurrence-free survival of 70.7% and 71.7% in the fluorescence and conventional surgery groups, respectively.

## 4. Discussion

To our knowledge, this study provides the largest systematic review and meta-analysis assessing the efficacy of fluorescence-guided surgery, along with a comparison to conventional surgery, in the management of CRC. The pooled analysis of fluorescence-guided surgery studies (including ICG, cRGD-ZW800-1, and SGM-101) demonstrated that fluorescence-guided surgery has a high cancer detection rate (71%) and lymph node yield ratio (42%), with a subsequent low complication rate (5%) and anastomotic leak rate (3%). There was no difference in the operative time and length of stay when comparing fluorescence-guided surgery with conventional surgery. Intraoperative blood loss was reduced, along with a 4% lower complication rate following fluorescence-guided surgery, compared to conventional surgery. One plausible reason for this is that the lymph node mapping aligned with the colon’s vascular architecture potentially enhanced the distinction between vessels designated for resection from those to be conserved. This precision may have minimised superfluous ischaemic regions. The conventional surgery group had a higher anastomotic leak rate (5%) and a lower lymph node yield ratio than the fluorescence-guided surgery group in our analysis; however, this was not found to be statistically significant. It is important to highlight that in studies with lymph node mapping, the NIRF camera was more often employed for perfusion assessment before the creation of the anastomosis, as compared to conventional surgeries, which may act as a confounding factor.

The application of ICG is now established in CRC surgery due its fluorescence emission properties. It has also been hypothesised that it can serve as a photosensitiser for photodynamic therapy applications, eliciting cytotoxic effects both in vitro and in vivo when used in combination with light at wavelengths in the region of 800–830 nm [55,56]. Recently, there has also been a growing increase in the number of tumour-specific targeted agents in CRC surgery. SGM-101 and bevacizumab-800CW are examples of monoclonal antibodies that target carcinoembryonic antigen (CEA) and vascular endothelial growth factor A (VEGF-A), respectively [44,47,57]. ONM-100 is a fluorescent agent that is activated in the acidic tumour microenvironment, and cRGD-ZW800-1 targets several integrins that have been shown to be overexpressed on colorectal tumour cells [58]. The action of these agents is to potentially enhance the detection of tumour tissue, reducing the number of tumour-positive resection margins, and avoiding the unnecessary removal of benign tissue [8]. In locally advanced rectal cancer, metastasis to lateral pelvic lymph nodes (common iliac, internal iliac, external iliac, and obturator nodes) can occur. Due to this reported high incidence (18.1%), the Japanese Society for Cancer of the Colon and Rectum (JSCCR) position total mesorectal excision (TME) and lateral lymph node dissection (LLND) as the standard of care. The JCOG0212 RCT, which compared TME with and without LLND in lower rectal cancer (stages II and III), showed local recurrence of 7.4% with TME/LLND versus 12.6% with TME only (*p* = 0.24). Local recurrence was mostly represented as lateral pelvic recurrence (1.1% TME/LLND versus 6.6% TME only) [58].

A retrospective study by Watanabe et al. [28] compared the long-term outcomes of fluorescence-guided and conventional laparoscopic lateral pelvic lymph node dissection in 116 CRC patients (after propensity score matching). The three-year cumulative lateral local recurrence was 0% in the fluorescence-guided surgery group versus 9.3% in the conventional surgery group. In addition, a retrospective study in 462 CRC patients (after propensity score matching) found a significantly higher number of total, intermediate, and central lymph nodes dissected in the fluorescence-guided surgery group compared to the non-fluorescence-guided surgery group [22]. However, there was no difference in mid-term oncological outcomes between the two groups. As highlighted by our systematic review and meta-analysis, the benefit of fluorescence-guided surgery on clinical endpoints, such as tumour-negative resection margin rates, detection of occult lesions, overall survival, and disease-free survival, is less clear and requires further investigation.

Nevertheless, fluorescence agents, such as ICG, have the potential to reduce the risk of anastomotic leakage by behaving as a predictive test of normal intestinal blood supply [59]. The perfusion assessment in laparoscopic left-sided/anterior resection (PILLAR II) study demonstrated insufficient blood circulation with the use of ICG in 6.5% of patients, in whom sufficient circulation was indicated in macroscopic evaluation [60]. There was no anastomotic leakage in the cases with alteration of the surgical plan after fluorescence angiography. However, the PILLAR III study found no difference in anastomotic leak rates between patients who underwent perfusion assessment versus the standard surgical technique, which compares to our findings in this systematic review and meta-analysis [61]. This is contrast to the FLAG study, which found a significant reduction in the anastomotic leak rate following anterior resection (14.4% in the fluorescence-guided surgery group vs. 25.7% in the non-fluorescence-guided surgery group) [62]. A meta-analysis of 554 patients showed a significant reduction in the anastomotic insufficiency rate after the use of ICG fluoroscopy, compared with conventional interventions (1.1% versus 6.1%, respectively), and an 81% anastomotic insufficiency reduction as a result of circulation assessment [59]. In addition, a study by Kudszus et al. [63] revealed a lower rate of reoperation following intraoperative use of fluoroscintigraphy compared to the control group (3.5% versus 7.5%, respectively). In the EssentiAl RCT, 839 patients were subject to ICG-guided surgery and conventional surgery for rectal cancer across 41 hospitals in Japan. They discovered that ICG significantly reduced the anastomotic leakage rate by 4.2% [54]. An unblinded, multicentre RCT (IntAct study) is currently underway, which will compare the anastomotic leak rate following surgery with intraoperative fluorescence angiography against conventional surgery [64]. Here, 880 patients will be recruited from 25 European centres over three years, and the patients will be followed up for 90 days.

The European Association for Endoscopic Surgery (EAES) consensus on ICG fluorescence-guided surgery recommended the use of ICG in colorectal surgery to assess tissue perfusion in order to reduce the risk of anastomotic leak [65]. The results from the AVOID study (NCT04712032), a phase III, randomised controlled trial that includes 978 patients, are currently pending [66]. The primary endpoint is the clinically relevant anastomotic leak, comparing two cohorts: the fluorescence-guided bowel anastomosis group and the conventional bowel anastomosis group. However, despite significant research in the field, a uniform quantitative fluorescence method is still lacking. This represents an obstacle, as there are no reproducible and comparable data in clinical studies. To have a thorough understanding of fluorescence and the need of quantification, the technical factors of fluorescence-guided surgery should be considered. First, fluorescence intensity is inversely correlated to the source-to-target distance, where a region of interest observed closely may appear more fluorescent than regions identified farther away from the camera lens. Second, perfusion fluorescence is a dynamic process; over time, ICG tends to diffuse even to ischaemic areas, thus overestimating such areas and making fluorescence intensity unreliable [67]. As a result, the use of quantitative methods, such as the fluorescence-based enhanced reality (FLER) software, allows for the accurate evaluation and display of the dynamic evolution of the fluorescent signal during angiography, thereby accurately assessing tissue perfusion [68]. Using quantitative approaches in fluorescence angiography may constitute a promising tool for establishing reproducible data in fluorescence-guided research.

There are other emerging intraoperative imaging techniques that may complement ICG and fluorescein-angiography, particularly in the assessment of anastomotic insufficiency. For example, hyperspectral imaging (HSI) is an optical imaging technique that uses light to assess tissue perfusion, including the water content, oxygenation, or haemoglobin content [69]. Jansen-Winkeln et al. [70] applied HSI along with ICG in 32 patients during colorectal resection and illustrated that both modalities can equally determine the perfusion border zone. This technique has been further optimised by Pfahl et al. [71], who developed and tested the first combined HSI and ICG system. Laser speckle contrast imaging (LSCI) is another promising technique that visualises microcirculatory tissue blood perfusion [72]. Nwaiau et al. [73] carried out a multi-modal LSCI and ICG perfusion assessment in 17 patients undergoing elective colectomy with anastomosis, along with 23 patients that had Roux-en-Y gastric bypass and sleeve gastrectomy. The authors demonstrated that the dual-mode LSCI and ICG imaging device is safe and can provide accurate, real-time continuous assessment of tissue perfusion. Further research is required to determine the exact benefit of these novel imaging techniques in the oncological clinical setting.

### Strengths and Limitations

A strength of this study is that it included a considerable sample size of 3217 patients that underwent fluorescence-guided and conventional surgery for CRC. Nevertheless, this study had important limitations that should be addressed. Between-study heterogeneity was apparent, particularly when analysing tumour size, estimated blood loss, and the cancer detection rate. In general, the majority of the studies were observational studies and presented data on fluorescence oncological outcomes only, rather than providing a direct comparison between fluorescence and conventional surgery groups. There were only five RCTs included, and not all the studies assessed every aspect reviewed by this meta-analysis. For example, it was not possible to compare oncological outcomes between fluorescence-guided surgery and conventional surgery groups, other than the lymph node yield ratio, due to a lack of comparative studies and data. Furthermore, there was also significant variation found in the length of follow-up between the included studies.

In addition, fluorescence-guided surgery is a relatively novel procedure, which some surgeons may be technically invested in, which will ultimately lead to the publication of optimal outcomes and underreporting of complications. The injectable costs and imaging system costs could also not be evaluated amongst studies. In other gastrointestinal domains, fluorescence-guided surgery has proven to be cost-effective, favouring the routine use of fluorescent cholangiography during laparoscopic cholecystectomy [74]. Considering the important economic societal burden of anastomotic leakage, estimated between EUR 1.9 and 6.1 million, it would be important to evaluate the cost-effectiveness in the use of fluorescent dyes in CRC studies [75].

## 5. Conclusions

The findings of this systematic review and meta-analysis have confirmed that fluorescence-guided surgery is a safe and effective approach. It can potentially lead to a high cancer detection rate and lymph node yield ratio. It led to a reduction in blood loss and overall complication rates when compared to conventional surgery, with some evidence to suggest it may lower rates of anastomotic leakage. However, fluorescence-guided surgery did not significantly improve the lymph node yield ratio when compared to conventional surgery, and the prognostic benefit remains uncertain. Further RCTs directly comparing fluorescence and conventional surgery are essential to determine whether fluorescence-guided surgery has any prognostic significance and to understand its exact role in the surgical management pathway of CRC.

## Figures and Tables

**Table 1:** Study design, patient characteristics, and quality assessment of the studies included in this systematic review and meta-analysis. AFI, autofluorescence imaging; CEA, carcinoembryonic antigen; FC, fluorescence camera; FIS, fluorescence imaging system; FGI, fluorescence-guided imaging; FS, fluorescence spectroscopy; GA, general anaesthesia; HRE, high-resolution white light endoscopy; ICG, indocyanine green; IV, intravenous; NIR, near-infrared; NIRF, near-infrared fluorescence; SLN, sentinel lymph node; VEGF-A, vascular endothelial growth factor A; - = not reported; N/A = not applicable; * = high-quality studies based on the Newcastle–Ottawa Scale (NOS).

Author, Year	Country	Study Design (Level of Evidence)	Sample Size, n	Median/Mean Age (Range/SD)	Sex, Males	Median/Total Follow-up (Range)	T1, n (%)	T2, n (%)	T3, n (%)	T4, n (%)	Type of Fluorescence Agent	How Fluorescence Agent Was Administered	Fluorescence Dose	Molecular Target	Imaging Technique/Device	Time between Injection and Imaging	NOS
de Gooyer et al. [30], 2022	Netherland	RCT (1)	15	-	4	-	-	-	-	-	111In (In-DOTA-labetuzumab-IRDye800CW)	IV	2 mg, 10 mg, 50 mg	CEA	NIR-FIS (Quest Medical Imaging Spectrum NIR FC))	5 or 6 days	6
de Valk et al. [32], 2020	Netherlands	RCT (1)	23	66.8 (49–80)	10 (0.434)	-	-	-	-	-	cRGD-ZW800-1	IV	0.001 and 0.005 mg/kg	Integrins	Olympus Visera Elite II (CLV-S200-IR)	2 to 4 h	N/A
Rotondano et al. [37], 2012	Italy	RCT (1)	47	AFI: 54 (15) and HRE: 51 (12)	AFI: 24 (0.51) and HRE: 21 (0.446)	-	-	-	-	-	-	-	-	N/A	Olympus XCF-H260AZI	-	N/A
Wan et al. [27], 2022	China	RCT (1)	66 (33 ICG vs. 33 non-ICG)	58 (39–79)	45	-	0	6 (0.09)	33 (0.5)	27 (0.41)	ICG	Endoscopic submucosal injection	2.5 mg/1 mL	N/A	FIS (Karl Storz)	1 day	N/A
Watanabe et al. [41], 2017	Japan	RCT (1)	31	67.5 (±12.2)	22 (0.80)	-	-	-	-	-	ICG	Laparoscopically injected	2.5 mg	N/A	Laparoscopic NIR camera system (Karl Storz)	30 min	N/A
Watanabe et al. [54], 2020	Japan	RCT (1)	839 (422 ICG vs. 417 non-ICG)	ICG: 66 (56–73) and non-ICG: 67 (58–74)	ICG: 266 (0.63) and non-ICG: 274 (0.66)	1 month	150 (0.36)	117 (0.28)	154 (0.37)	-	ICG	IV	12.5 mg	N/A	Endoscopic camera system 1588 and 1688 (Stryker Corporation)	1 min	N/A
Ankersmit et al. [34], 2019	Netherlands	Prospective (2)	10	71.5 (63–77)	8	-	-	-	-	-	[89Zr]Zr-Nanocoll (preoperative); ICG (intraoperative)	Endoscopically	“89Zr 0.4 mL	N/A	PET/CT; NIR laparoscopy (Olympus)	46 (43–48) h	5
Cai et al. [29], 2023	China	Prospective (2)	34	55.7 (±9.6)	22	-	-	-	-	-	ICG	IV	0.5 mg/kg	N/A	Laparoscopic FIS (Stryker Corporation)	5–7 days	6
Chand et al. [20], 2018	UK	Prospective (2)	10	69.5 (±7.13)	4	30 days	1 (0.1)	0 (0)	3 (0.3)	6 (0.6)	ICG	1 mL subserosal ICG injection placed in four sites around the tumour	Varying concentrations (5 mg/10 mL, 5 mg/5 mL, 5 mg/3 mL)	N/A	Laparoscopic FC systems (Pinpoint and AIM 1588)	Immediate (intraoperative)	7 *
Currie et al. [22], 2017	UK	Prospective (2)	30	68 (38–80)	-	-	6 (0.2)	8 (0.27)	14 (0.47)	2 (0.067)	ICG	Four 1 mL aliquots around the tumour	5 mg/mL	N/A	Laparoscopic NIR FIS (Olympus)	Immediate (intraoperative)	7 *
de Valk et al. [47], 2021	Netherlands	Prospective (2)	37	63 (±8.7, 43–79)	23 (0.62)	Immediate (postoperative)	-	-	-	-	SGM-101	IV over 30 min	5 mg, 7.5 mg, 10 mg, 12.5 mg, 15 mg	CEA	Quest Spectrum Platform (Quest Medical Imaging)	At least 24 h	7 *
Hellan et al. [23], 2014	USA	Prospective (2)	40 (28 with CRC)	63.9	20	30 days	-	-	-	-	ICG	IV	Max dose 2 mg/kg	N/A	Fluorescence-capable da Vinci Si high-definition vision system (Firefly)	Mean 5.1 (+/−10) min	7 *
Hirche et al. [24], 2012	Germany	Prospective (2)	26	67 (46–87)	-	-	6 (0.23)	5 (0.19)	14 (0.54)	1 (0.038)	ICG	Intraoperatively around the tumour	5 mg/mL	N/A	FIS (IC-View) consisting of digital video camera with an integrated NIR light source	3–10 min	7 *
Kim et al. [33], 2020	Korea	Prospective (2)	10	60 (48–80)	8	-	-	-	-	-	ICG	Transanal	2.5 mg/bodyweight	N/A	da Vinci Surgical System (Intuitive Surgical)	Immediate (intraoperative)	5
Kinoshita et al. [46], 2023	Japan	Prospective (2)	56	74 (65–77)	35 (0.625)	-	13 (0.232)	10 (0.179)	23 (0.411)	10 (0.179)	ICG	Injection to subserosal layer at both proximal and distal points of the tumour	0.2−0.5 mL at a concentration of 2.5 mg/mL	N/A	1588 AIM camera system; infrared endoscopic camera system; D-Light P (Karl Storz)	5 min, 30–60 min, and over 60 min	7 *
Miyoshi et al. [35], 2009	Japan	Prospective (2)	40	63.5 (41–84)	22 (0.55)	>9 days	-	-	-	-	ICG	-	-	N/A	CF-FH260AZI endoscope	-	7 *
Moriichi et al. [36], 2012	Japan	Prospective (2)	67	-	-	-	-	-	-	-	-	-	-	N/A	AFI	-	6
Munechika et al. [50], 2021	Japan	Prospective (2)	40 (20 ICG vs. 20 non-ICG)	68.5 (±8.3, 47–83)	36 (0.65)	15 months (6–22)	21 (0.525)		19 (0.475)		ICG	IV	5 mg	N/A	ICG NIRF (1588 AIM camera system, Stryker Corporation)	24 s (3-61)	
Patel et al. [31], 2022	UK	Prospective (2)	15	-	7	-	-	-	-	-	ICG	IV	10 mg/kg bodyweight	N/A	NIRF (IMAGE1 Spies, Karl Storz)	1 day	5
Peloso et al. [38], 2013	Italy	Prospective (2)	25	61.5 (42–87)	17 (0.68)	-	-	-	-	-	ICG	IV	0.5 mg/kg bodyweight	N/A	NIR FC, the Photodynamic Eye (PDE) (Hamamatsu Photonics))	24 h	7 *
Sato et al. [51], 2023	Japan	Prospective (2)	14	71.2 (±11.7,42–87)	8 (0.57)	Immediate (postoperative)	2 (0.14)	3 (0.21)	6 (0.43)	3 (0.21)	ICG	0.1 (0.25 mg) of ICG injected into submucosa at the dentate line 3 cm from the anal verge at the anterior, posterior, and bilateral walls	2.5 mg/mL (25 mg in 10 mL distilled water)	N/A	Laparoscopic NIRF system (VISERA ELITE II, Olympus)	Immediate	7 *
Schaafsma et al. [25], 2013	USA	Prospective (2)	22	69 (41–88)	12	-	2 (0.09)	7 (0.32)	10 (0.45)	3 (0.14)	HSA800 and blue dye	Injected submucosally circumferentially with a 5 mm margin around the tumour	1 mL of 50 micromol/L HSA800 diluted in patent blue dye	N/A	Mini-FLARE camera system	5 min	7 *
Schaap et al. [52], 2020	Netherlands	Prospective (2)	14	-	-	Immediate (postoperative)	-	-	-	-	SGM-101	IV	-	CEA	Quest Spectrum Platform (Quest Medical Imaging)	4–6 days before surgery	7 *
Sikkenk et al. [26], 2023	Netherlands	Prospective (2)	10	70 (59–84)	7	-	5 (0.5)		0	0	ICG	1 mL ICG in four aliquots, injected submucosally around the tumour	5 mg/ml	N/A	NIRF ‘firefly’ mode of da Vinci Xi	Immediate (intraoperative)	7 *
Tashiro et al. [32], 2020	Japan	Prospective (2)	72	67.5 (34–85)	23	-	-	-	-	-	ICG	IV	0.5 mg/kg	N/A	SPY Portable Handheld Imaging System (Stryker Corporation)	5 (2–34) days	6
Tanis et al. [39], 2016	Netherlands	Prospective (2)	17	62 (38–74)	13 (0.764)	-	-	-	-	-	-	Optical needle	-	N/A	Diffuse reflectance spectroscopy and FS	-	6
Weixler et al. [42], 2017	Switzerland	Prospective (2)	220	70.5 (±11.2)	126 (0.573)	73.3 months (70.4–76.2)	20 (0.91)	37 (0.168)	149 (0.677)	14 (0.44)	ICG	Ex vivo injected with ICG subserosa around the tumour	-	N/A	ICG-based SLN-mapping	5 min	8 *
Daibo et al. [22], 2024	Japan	Retrospective (3)	462 (231 ICG vs. 231 non-ICG after propensity scoring)	75 (68–79)	216	36.9 months	140 (0.30)	92 (0.20)	228 (0.49)	0	ICG	Subserosal submucosal layer injection around the tumour at two points	2.5 mg/ml	N/A	ICG-FI systems (D-light, Karl Storz)) and endoscopic camera system (Stryker Corporation)	30 min	9*
de Jongh et al. [43], 2020	Netherlands	Retrospective (3)	25	56 (31–76)	17 (0.68)	-	-	-	-	-	Bevacizumab-800CW	Back-table FGI	-	VEGF-A	High-resolution Odyssey CLx FIS (LI-COR Biosciences Inc.)	-	7 *
Handgraaf et al. [40], 2017	Netherlands	Retrospective (3)	173 (106 fluorescence vs. 67 non-fluorescence)	Non-fluorescence: 63 (±9.4) vs. fluorescence: 62 (±9.2)	94 (0.54)	4 years	-	-	-	-	ICG	IV	10 or 20 mg	N/A	Mini-FLARE^®^ (Frangioni Laboratory), Artemis (Quest Innovations)), and laparoscope (Karl Storz)	1 or 2 days	9 *
Mizrahi et al. [48], 2018	USA	Retrospective (3)	54	63 (±12)	31 (0.57)	Immediate (postoperative)	5 (0.09)	14 (0.26)	28 (0.52)	2 (0.04)	-	IV 3.5 mL ICG followed by a second and third bolus of same volume	2.5 mg/mL (25 mg in 10 mL sterile water)	N/A	PINPOINT endoscopic FIS (Novadaq))	Immediate (intraoperative)	7 *
Mizrahi et al. [49], 2018	USA	Retrospective (3)	60 (30 fluorescence vs. 30 non-fluorescence)	58 (±12)	34 (0.57)	Immediate (postoperative)	8 (0.13)	10 (0.167)	20 (0.33)	2 (0.033)	ICG	IV 3.5 mL ICG followed by a second and third bolus of same volume	2.5 mg/mL (25 mg in 10 mL sterile water)	N/A	PINPOINT endoscopic FIS (Novadaq))	Immediate (intraoperative)	8 *
Park et al. [45], 2020	Korea	Retrospective (3)	75 (25 ICG vs. 50 non-ICG)	ICG:71 (49–83) and non-ICG: 66 (42–84)	42 (0.56)	Immediate (postoperative)	-	-	58 (0.77)	17 (0.23)	ICG	ICG solution was injected into the submucosa in the peritumoral area at one or two points	0.2–0.3 mL of 2.5 mg/mL	N/A	D-light P (Karl Storz)and Firefly (Intuitive Surgical)	3 to 24 h	8 *
Watanabe et al. [53], 2020	Japan	Retrospective (3)	422 (211 ICG vs. 211 non-ICG after propensity scoring)	66 (34–92)	259 (0.61)	Immediate (postoperative)	-	-	-	-	ICG	IV	0.25 mg/kg	N/A	NIR camera system	Immediate—just before proximal bowel resection	8 *
Watanabe et al. [28], 2023	Japan	Retrospective (3)	116 (58 ICG vs. 58 non-ICG after propensity matching)	65 (60–73)	78	63.7 months (51.3–76.8)	23 (0.2)	40 (0.34)	48 (0.41)	0	ICG	Injected locally into submucosal layer at four points (total 1 mL) on anal side of tumour using a flexisigmoidoscopy	2.5 mg/1 mL	N/A	Laparoscopic NIRF system, 1588 AIM (Stryker Corporation) and D-light (Karl Storz)	Injected after GA induction	9 *

## Data Availability

The datasets generated and analysed during the current study are available from the corresponding author upon request.

## Supplementary Materials

The following supporting information can be downloaded at: https://www.mdpi.com/article/10.3390/cancers16193377/s1, Table S1. Systematic review search strategy created for MEDLINE, EMBASE, Emcare and CINAHL databases between 1 January 2000 and 1 January 2024. Table S2. Newcastle-Ottawa scale scoring of observational studies included in the systematic review. * = Newcastle-Ottawa scale not applicable as randomised controlled trial. Table S3. Inclusion and exclusion criteria of studies in systematic review. ASA, American Society of Anesthesiologists physical status classification system; CEA, carcinoembryonic antigen; CRLM, colorectal liver metastases; ICG, indocyanine green; LAR, low anterior resection. LPLND, laparoscopic lymph node dissection; LPN, lateral pelvic lymph node; MELD, Model for End-Stage Liver Disease; TaTME, trans-anal total mesorectal excision; NIRF, near-infrared fluorescence; TPE, total pelvic exenteration; - = not reported.
