# Supplementary material for: Efficacy and Safety of Fluorescence-Guided Surgery Compared to Conventional Surgery in the Management of Colorectal Cancer: A Systematic Review and Meta-Analysis
Source: Cancers (Basel). 2024 Oct 2;16(19):3377. doi: 10.3390/cancers16193377 (PMC11476237; doi:10.3390/cancers16193377)
Supplement: Supplementary file 1 [file cancers-16-03377-s001.zip › cancers-3161624-supplementary.pdf]

Supplementary material

Search	Query	Results
1	(medical optical imaging or spectroscopy or endoscopy or laser doppler imaging or optical coherence tomography).	709599
2	exp Optical Imaging/ or spectrum analysis/ or spectrometry, fluorescence/ or exp Endoscopy/	586715
3	1 or 2	1152302
4	flourescence	504483
5	Fluorescence/ or Fluorescence	46769
6	4 or 5	504483
7	3 and 6	118606
8	(near-infrared spectroscopy or NIRS)	17706
9	(Diffuse optical imaging or diffuse optical tomography or DOI or DOT)	76178
10	(Near-infrared optical tomography or fluorescence diffuse optical tomography or NIROT or FDOT)	150
11	Spectroscopy, Near-Infrared/	17562
12	tomography, optical/ or tomography, optical coherence/	101735
13	8 or 9 or 10 or 11 or 12	382
14	((fluorescence guided surgery and (open or laparoscopic)) or (fluorescence adj3 (open or laparoscopic)))	864
15	fluorescence guided surgery	93
16	((fluorescence guided surgery and (open or laparoscopic)) or (fluorescence guided surgery adj3 (open or laparoscopic)))	17428
17	(fluorescence angiography or fluorescence imaging)	16042
18	indocyanine green	80612
19	((near-infrared or fluorescence) and imaging)	53404
20	(fluores* angiography or fluores* imaging)	36918
21	Fluorescein Angiography/	124317
22	Indocyanine Green/	316126
23	(colorectal cancer or CRC)	133634
24	(((bowel or colon* or rect*) and (cancer or tumo?r* or neoplasm*)) or ((bowel or colon* or rect*) adj3 (cancer or tumo?r* or neoplasm*)))	338726
25	(bowel cancer or bowel tumo?r* or bowel neoplasm*)	3281
26	(colon cancer or colon tumo?r* or colon neoplasm*)	62370
27	(rectal cancer or rectal tumo?r* or rectal neoplasm*)	56594
28	colorectal neoplasms/ or exp colonic neoplasms/ or exp rectal neoplasms/	226716
29	23 or 24 or 25 or 26 or 27 or 28	440954
30	22 and 29	3033
31	exp Animals/	26281404
32	Humans/	21171465
33	31 not 32	5109939
34	30 not 33	2735
35	limit 34 to (english language and humans and yr="2000 -Current")	1780
36	35 and "Research Support, Non-U.S. Gov't".sa_pubt.	802
37	35 and "Research Support, N.I.H., Extramural".sa_pubt.	186
38	35 and "Letter".sa_pubt.	47
39	35 and "Video-Audio Media".sa_pubt.	74
40	35 and "Case Reports".sa_pubt.	110
41	35 and "Congress".sa_pubt.	1
42	35 and "Research Support, N.I.H., Intramural".sa_pubt.	8
43	35 and "Research Support, U.S. Gov't, Non-P.H.S.".sa_pubt.	53
44	35 and "Research Support, U.S. Gov't, P.H.S.".sa_pubt.	22
45	35 and "Retracted Publication".sa_pubt.	2
46	36 or 37 or 38 or 39 or 40 or 41 or 42 or 43 or 44 or 45	1054
47	35 not 46	726
48	meta-analysis/ or "systematic review"/	306889
49	exp clinical trial/ or exp randomized controlled trial/	967252
50	48 or 49	1273360
51	47 and 50	59
52	limit 47 to (meta analysis or "review" or "systematic review")	89
53	limit 47 to (clinical study or clinical trial, all or clinical trial, phase i or clinical trial, phase ii or clinical trial, phase iii or clinical trial, phase iv or clinical trial or comparative study or	102

	controlled clinical trial or equivalence trial or evaluation study or multicentre study or observational study or pragmatic clinical trial or randomized controlled trial)	
54	52 or 53	187
55	51 or 54	187

Table S1. Systematic review search strategy created for MEDLINE, EMBASE, Emcare and CINAHL databases between 1st January 2000 and 1st January 2024.

Author, year	Selection				Comparability		Outcome			Total score
	Representativeness of the exposed cohort	Selection of the non- exposed cohort	Ascertainment of exposure	Outcome not present at start	Comparability of cohorts on the basis of the design or analysis	Study controls for additional factor	Assessment of outcome	Was follow-up long enough	Adequacy of follow up	
Chand et al. [20], 2018	☆		☆	☆	☆	☆	☆		☆	7
Currie et al. [21], 2017	☆		☆	☆	☆	☆	☆		☆	7
Daibo et al. [22], 2024	☆	☆	☆	☆	☆	☆	☆	☆	☆	9
Hellan et al. [23], 2014	☆	☆	☆	☆	☆	☆	☆			7
Hirche et al. [24], 2012	☆	☆	☆	☆	☆	☆	☆			7
Schaafsma et al. [25], 2013	☆	☆	☆	☆	☆	☆	☆			7
Sikkenk et al. [26], 2023	☆	☆	☆	☆	☆	☆	☆			7
Wan et al. [27], 2022	-	-	-	-	-	-	-	-	-	N/A*
Watanabe et al. [28], 2023	☆	☆	☆	☆	☆	☆	☆	☆	☆	9
Cai et al. [29], 2023	☆		☆	☆			☆	☆	☆	6
de Gooyer et al. [30], 2022	☆		☆	☆			☆	☆	☆	6
Patel et al. [31], 2022	☆		☆	☆				☆	☆	5
Tashiro et al. [32], 2020	☆		☆	☆			☆	☆	☆	6
Kim et al. [33], 2020	☆		☆	☆				☆	☆	5
Ankersmit et al. [34], 2019	☆		☆	☆				☆	☆	5
Miyoshi et al. [35], 2009	☆		☆	☆	☆	☆	☆	☆		7
Moriichi et al. [36], 2012	☆		☆	☆		☆	☆	☆		6
Rotondano et al. [37], 2012	-	-	-	-	-	-	-	-	-	N/A*
Peloso et al. [38], 2013	☆		☆	☆	☆	☆	☆	☆		7
Tanis et al. [39], 2016	☆		☆	☆		☆	☆	☆		6
Handgraaf et al. [40], 2017	☆	☆	☆	☆	☆	☆	☆	☆	☆	9
Watanabe et al. [41], 2017	-	-	-	-	-	-	-	-	-	N/A*
Weixler et al. [42], 2017	☆		☆	☆	☆	☆	☆	☆	☆	8
de Jongh et al. [43], 2020	☆		☆	☆	☆	☆	☆	☆		7

de Valk et al. [44], 2020	-	-	-	-	-	-	-	-	-	N/A*
Park et al. [45], 2020	☆	☆	☆	☆	☆	☆	☆	☆		8
Kinoshita et al. [46], 2023	☆		☆	☆	☆	☆	☆	☆		7
de Valk et al. [47], 2021	-	-	-	-	-	-	-	-	-	7
Mizrahi et al. [48], 2018	☆		☆	☆	☆	☆	☆	☆		7
Mizrahi et al. [49], 2018	☆	☆	☆	☆	☆	☆	☆	☆		8
Munechika et al. [50], 2021	☆	☆	☆	☆	☆	☆	☆	☆		8
Sato et al. [51], 2023	☆		☆	☆	☆	☆	☆	☆		7
Schaap et al. [52], 2020	☆		☆	☆	☆	☆	☆	☆		7
Watanabe et al. [53], 2020	☆	☆	☆	☆	☆	☆	☆	☆		8
Watanabe et al. [54], 2023	-	-	-	-	-	-	-	-	-	N/A*

Table S2. Newcastle-Ottawa scale scoring of observational studies included in the systematic review. * = Newcastle-Ottawa scale not applicable as randomised controlled trial.

Author, year	Inclusion criteria	Exclusion criteria
Chand et al. [20], 2018	18 years or over, diagnosed with primary colon cancer proximal to rectosigmoid junction, scheduled for curative surgical resection of cancer	Under 18, presenting with bowel obstruction, undergoing emergency/urgent resection or surgery for palliative intent, distant metastasis, allergy or history of adverse reaction to ICG/iodine, hepatic (MELD score >10) or renal dysfunction (Creatinine level over 2 mg/dl)
Currie et al. [21], 2017	Patient preoperatively staged as T1/T2 colonic cancer on CT and suitable for laparoscopic colonic resection	Patients with suspected T3 or T4 colon cancer, pregnancy, allergy to ICG, iodine, patients with hyperthyroidism/thyroid adenomas or renal dysfunction (Creatinine > 110 mg/dl)
Daibo et al. [22], 2024	Laparoscopic right-sided colectomy for colon cancer - 18 years or older, colon cancer in caecum, ascending colon or right-sided transverse colon, laparoscopic colectomy with D3 lymph node dissection or staging 1-3	Open or robotic surgery, other surgery at same time, adjacent organs resected, iodine allergy
Hellan et al. [23], 2014	Patients who underwent robotic left-sided colon or rectal resections for benign or malignant indications	-
Hirche et al. [24], 2012	Histopathological diagnosis of colon cancer, image-guided tumour diameter without distinct evidence for serosal perforation, and exclusion of clinically and image-guided positive lymph node status	Previous operations/radiation, definite lymph node metastases diagnosed preoperatively, preoperative tumour stage T4, pregnancy, age <18 years
Schaafsma et al. [25], 2013	Curative intent for colorectal cancer	Metastatic disease
Sikkenk et al. [26], 2023	cT1-2N0M0 colon cancer, age 18 years or over	Prior local excision of the tumour, suspicion of lymph node or distant metastases, contraindication for robot-assisted surgery or use of ICG (iodine allergy, severe kidney or liver failure, hyperthyroidism), pregnancy and lactation, tumour too large to pass endoscopically, metastatic disease or T4 tumour discovered during intraoperative staging
Wan et al. [27], 2022	18-80 years, ASA 1-3, single sigmoid or rectal cancer confirmed by endoscopic biopsy, planed laparoscopic radical resection	Previous abdominal tumour surgery, pregnancy and lactation, emergency patients with obstruction or perforation, T4b cancer evaluated by CT, MRI or EUS, pelvic or distant metastasis, T1 cancer planned for local excision, allergy, preoperative neoadjuvant therapy and low rectal cancer with lateral pelvic lymph node metastasis
Watanabe et al. [28], 2023	20 years or over with rectal cancer, histologically proven adenocarcinoma, signet cell or mucinous carcinoma, clinical stage II-III, lower edge of tumour within 100mm of AV, patient underwent laparoscopic LPLND	Preoperatively diagnosed with distant metastasis, underwent TPE, open or robotic surgery, patients who required resection at two or more primary sites and have iodine allergy
Cai et al. [29], 2023	86 patients with hepatic malignancies including hepatocellular carcinoma and CRLM	-
de Gooyer et al. [30], 2022	18 years of age or older, had histologically proven peritoneal metastases of colorectal cancer	Patients were excluded from participation if they were pregnant or breastfeeding, had a serum CEA concentration of >500 ng/ml, had a known CEA negative malignancy, if a radionuclide had been administered within 10 physical half-lives prior to study enrolment or if coinciding uncontrolled medical conditions were present
Patel et al. [31], 2022	Patients who were undergoing hepatectomy for superficial CRLM	Patients were excluded if they had a contrast allergy or could not be admitted to hospital the day before surgery (and therefore unable to receive a preoperative ICG injection)

Tashiro et al. [32], 2020	Patients who underwent liver resection for hepatocellular carcinoma or CRLM	-
Kim et al. [33], 2020	Patients underwent total mesorectal excision with LPND for rectal cancers after preoperative CRT. LPND was indicated in patients who were diagnosed with suspicious LPNs that were metastasized, defined as "index LPNs," based on a pretreatment radiologic examination (diameter of >5 mm with MRI)	-
Ankersmit et al. [34], 2019	Patients were eligible if at least 18 years of age and scheduled for a laparoscopic resection of a histopathologically proven colon carcinoma or suspected malignant lesion seen during colonoscopy.	-
Miyoshi et al. [35], 2009	Patients who underwent either laparoscopic or open colorectal surgery	History of allergy to iodinated contrast material
Moriichi et al. [36], 2012	Patients who underwent colonoscopy	-
Rotondano et al. [37], 2012	Personal history of neoplasia (adenoma or cancer) or a positive family history for colorectal cancer	Age younger than 18 years, polyposis syndromes, inflammatory bowel disease, severe coagulopathy and insufficient bowel preparation
Peloso et al. [38], 2013	Patients with liver metastases from primary colorectal cancers	Allergies to iodine or ICG
Tanis et al. [39], 2016	Patients ≥18 years old scheduled for an open liver resection for metastatic colorectal cancer	Patients who had no residual disease after neo-adjuvant chemotherapy or who had suspected sensitivity to light
Handgraaf et al. [40], 2017	Patients undergoing resection of CRLM with or without NIRF imaging	Contraindications for ICG: eGFR < 55; pregnancy; breastfeeding; hyperthyroidism; or an allergy to iodine, shellfish, or ICG.
Watanabe et al. [41], 2017	Lymph node with a minor axis measuring >10 mm on CT	A past history of colonic surgery, extended colorectal resection, allergic hypersensitivity to ICG, or allergic hypersensitivity to iodine
Weixler et al. [42], 2017	Stage I–III colon cancer patients	AJCC stage IV, age \18 years, extraperitoneal rectal cancer, prior abdominal cancer surgery, history of other malignancies, allergy to isosulfan blue or ICG, pregnancy, and breast-feeding
de Jongh et al. [43], 2020	Histologically proven locally advanced rectal cancer, with the inferior margin within 16 cm from the anal verge, and treatment with long-course neoadjuvant chemoradiotherapy	-
de Valk et al. [44], 2020	Phase I study in healthy volunteers and a phase II study in patients with colon cancer.	-
Park et al. [45], 2020	Patients who underwent colon cancer surgery	Patients with colonoscopic tumour removal, a clinical stage T1 or T2 tumour on the CT scan, open surgery, distant metastasis, an emergent operation, or palliative resection
Kinoshita et al. [46], 2023	Patients who underwent complete mesocolic excision and central vascular ligation for colon cancer	History of ICG or iodine allergies
de Valk et al. [47], 2021	Patients with primary and recurrent colorectal cancer undergoing surgery	Peritoneal metastasis, pancreatic cancer, liver metastasis
Mizrahi et al. [48], 2018	Patients who had a hybrid transabdominal LAR and TaTME for rectal cancer between July 2015 and May 2017 with a colorectal or coloanal anastomosis	Planned laparotomy, a redo coloanal anastomosis, an urgent or emergent operation, and/ or anastomosis >10 cm from the anal verge, or a planned abdominoperineal resection.
Mizrahi et al. [49], 2018	Patients who underwent elective laparoscopic LAR for a rectal neoplasm with a colorectal or coloanal anastomosis < 5 cm from the anal verge	Planned laparotomy, a redo coloanal anastomosis, an urgent or emergent operation, and/or anastomosis > 5 cm from the anal verge, or a planned abdominoperineal resection.

Munechika et al. [50], 2021	Patients with colon cancer located in the descending colon (including the sigmoid-descending colon junction) that underwent elective laparoscopic surgery	History of previous colorectal surgery and a history of hypersensitivity reaction to iodinated contrast media.
Sato et al. [51], 2023	Patients with rectal cancer who underwent laparoscopic or robot-assisted radical surgery, including cases in which TaTME was used in combination, without any preoperative therapy	Iodine hypersensitivity and those who did not consent
Schaap et al. [52], 2020	Patients diagnosed with peritoneal metastasis of colorectal origin	-
Watanabe et al. [53], 2020	Rectal cancer located within 15 cm from the anal verge with histologically proven adenocarcinoma or signet-ring cell carcinoma and having undergone laparoscopic LAR	Multiple primary cancers, a history of treatment for other pelvic malignancy, open or robotic surgery cases and emergent cases.
Watanabe et al. [54], 2023	Patients with histologically proven rectal cancer, a lower margin of the tumour less than 12 cm from the anal verge, clinically diagnosed as Union for International Cancer Control TNM classification (eighth edition) stage 0-III, scheduled for minimally invasive sphincter-preserving surgery with anastomosis [laparoscopic surgery, robotic surgery, or transanal total rectal resection (taTME) were eligible], older than 20 years of age, and Eastern Cooperative Oncology Group Performance status 0-2	Patients with allergies to iodine or ICG, patients planning to undergo surgery for other organ resection, and patients with serious comorbidity.

Table S3. Inclusion and exclusion criteria of studies in systematic review. ASA, American Society of Anesthesiologists physical status classification system; CEA, carcinoembryonic antigen; CRLM, colorectal liver metastases; ICG, indocyanine green; LAR, low anterior resection. LPLND, laparoscopic lymph node dissection; LPN, lateral pelvic lymph node; MELD, Model for End-Stage Liver Disease; TaTME, trans-anal total mesorectal excision; NIRF, near-infrared fluorescence; TPE, total pelvic exenteration; - = not reported.
